# Hepatitis A: Viral Structure, Classification, Life Cycle, Clinical Symptoms, Diagnosis Error, and Vaccination

**DOI:** 10.1155/2023/4263309

**Published:** 2023-01-04

**Authors:** Omid Gholizadeh, Sama Akbarzadeh, Mohamad Ghazanfari Hashemi, Marjan Gholami, Parya Amini, Zahra Yekanipour, Raheleh Tabatabaie, Saman Yasamineh, Parastoo Hosseini, Vahdat Poortahmasebi

**Affiliations:** ^1^Department of Bacteriology and Virology, Faculty of Medicine, Tabriz University of Medical Sciences, Tabriz, Iran; ^2^Infectious and Tropical Diseases Research Center, Tabriz University of Medical Sciences, Tabriz, Iran; ^3^Department of Animal Biology, Faculty of Natural Science, University of Tabriz, Tabriz, Iran; ^4^Department of Radiology, School of Medicine, Tehran University of Medical Sciences, Tehran, Iran; ^5^Department of Pharmacy, Faculty of Pharmacy, Islamic Azad University Pharmaceutical Sciences Branch, Tehran, Iran; ^6^Department of Microbiology, School of Medicine, Yasuj University of Medical Sciences, Yasuj, Iran; ^7^Department of Immunology, Medical Sciences University of Hamedan, Hamedan, Iran; ^8^Department of Virology, School of Public Health, Tehran University of Medical Sciences, Tehran, Iran; ^9^Research Center for Clinical Virology, Tehran University of Medical Sciences, Tehran, Iran

## Abstract

Hepatitis A virus (HAV) is one of the well-known viruses that cause hepatitis all around the globe. Although this illness has decreased in developed countries due to extensive immunization, numerous developing and under-developed countries are struggling with this virus. HAV infection can be spread by oral-fecal contact, and there are frequent epidemics through nutrition. Improvements in socioeconomic and sanitary circumstances have caused a shift in the disease's prevalence worldwide. Younger children are usually asymptomatic, but as they become older, the infection symptoms begin to appear. Symptoms range from slight inflammation and jaundice to acute liver failure in older individuals. While an acute infection may be self-limiting, unrecognized persistent infections, and the misapplication of therapeutic methods based on clinical guidelines are linked to a higher incidence of cirrhosis, hepatocellular carcinoma, and mortality. Fortunately, most patients recover within two months of infection, though 10–15% of patients will relapse within the first six months. A virus seldom leads to persistent infection or liver damage. The mainstay of therapy is based on supportive care. All children from 12–23 months, as well as some susceptible populations, should receive routine vaccinations, according to the Centers for Disease Control and Prevention and the American Academy of Pediatrics. Laboratory diagnosis of HAV is based on antigen detection, checking liver enzyme levels, and antibody screening. Furthermore, polymerase chain reaction (PCR) technology has identified HAV in suspected nutrition sources; therefore, this technique is used for preventative measures and food-related laws.

## 1. Introduction

Acute viral hepatitis is a systemic illness that mainly affects the liver. Hepatitis A virus (HAV), hepatitis B virus (HBV), hepatitis D virus (HDV), and hepatitis E virus (HEV) are the viruses that cause almost all instances of acute viral hepatitis [1]. Hepatitis can be present with little or no symptoms, although it frequently results in jaundice, anorexia, and malaise. Hepatitis infection is divided into two types: acute and chronic. Acute hepatitis remains for less than six months, whereas chronic hepatitis stays for an extended period [2].

In this perusal, we look at HAV, initially discovered in the feces of tentatively infected human prisoner volunteers using electron microscopy [3]. HAV infection is highly contagious [4], and it is a leading cause of acute hepatitis. In people with chronic liver disorders, acute HAV infection can induce liver failure [5]. HAV seroprevalence is also relatively low in high-income provinces. Socioeconomic factors, access to clean water, and proper sanitation are all strongly linked to epidemiology [6]. HAV usually causes short-term, self-limiting illnesses that go away without any long-term effects in 4–7 weeks. Dissimilar to HBV and HCV, HAV seems not to cause persistent liver damage. However, particularly in the elderly, severe fulminant hepatitis with possibly fatal liver failure may ensue. The associated risk factors that cause case-to-case variation in the degree of liver disease and recurrence remain unclear [7–10]. Furthermore, contact with the feces of infected patients with HAV can result in the transmission of the virus, implying that the virus can be spread via oral-fecal transmission [8, 11]. It is widespread, although it is more common in less-developed areas with inadequate sanitary and hygienic conditions [12]. Transfusion transmission of HAV is exceedingly rare due to the short persistence of viremia throughout acute HAV infection (approx. 10–50 days) [13]. However, donors should be reminded that if they are identified with blood-borne diseases after donating blood, they must inform the blood center. If a transfused individual shows hepatitis A symptoms with no record of traveling or oral-fecal disease, doctors should evaluate the potential of transfusion-transmitted hepatitis A [14].

Sporadic or epidemic outbreaks of HAV even now arise in several parts of the world, particularly in developing nations. In regions where people are mostly vaccinated, the prevalence of HAV has dropped considerably. In more industrialized nations, there is a growing movement toward vaccinating children, as this minimizes the chance of infection in the rest of the population. A prominent event occurs in countries with median endemicity when the disease is rare due to the amended water treatment and hygiene. However, grown-ups with no earlier contact become infected through sporadic outbreaks with clinically apparent symptoms [3, 15]. Symptoms may recur in up to 10 percent of patients after recovery, causing the disease to last for weeks or months [16]. Liver damage can be severe, especially in adults who lack protective antibodies acquired during childhood due to the virus's lower incidence. Hepatic failure that results in death happens in less than 1 percent of cases [3, 15]. Despite vaccine success, HAV continues to be a leading reason for enterically spreading hepatitis worldwide, causing epidemics in both developing and industrialized nations, with symptoms varying from moderate jaundice and inflammation to abrupt liver failure [17]. The scientists examined the duration of HAV being eliminated from the environment. Their findings suggest that HAV in fetal rhesus monkey kidney cell line (FRHK4)-infected cells may survive for more than 17 weeks and up to 10 weeks at room temperature, based on the size of needles and the volume of dead syringes on stoves, cotton/filter surfaces, and tourniquets. At a pH lower than 2, HAV may be infectious for more than ten weeks. Consequently, depending on the tested equipment and the environmental circumstances, HAV survives by utilizing medication injection tools and remains contagious for 1–10 weeks [18].

The purpose of this study is to examine the existing evidence for hepatitis A screening, diagnosis, and treatment. The structure of the HAV virus, the virus genome, how it is diagnosed, how it spreads, the importance of vaccination, and the factors derived from the HAV virus are all discussed.

## 2. HAV Rate

Every year, millions of HAV cases are recorded, with a more significant occurrence in underdeveloped countries [19]. The World Health Organization (WHO) estimates 1.5 million clinical cases of HAV per year. However, the true incidence of infection could be ten times higher, causing about 7,134 deaths in 2016, which represents 0.5% of the mortality attributable to viral hepatitis [20, 21]. After the introduction of hepatitis A vaccine recommendations in the United States in 1996, reports of HAV cases decreased progressively from 1999–2011 by ≈95%. However, researchers newly showed that hepatitis A cases boosted 294% during 2016–2018 compared with 2013–2015 among persons who use drugs (injection or noninjection), homeless people, or men who have sex with men (MSM). Also, Centers for Disease Control and Prevention (CDC) reports showed 3,366, 12,474, and 18,846 reported cases in 2017, 2018, and 2019, respectively [4, 22] (Table 1).

Over 90% of children experience the infection before the age of ten. HAV infection is asymptomatic in around 70% of kids under six, with the majority showing no clinical indications of liver disease, while symptoms appear in 70% of adolescents and adults. In adults, HAV can cause severe morbidity, with jaundice occurring in 40–70% of cases [13].

### 2.1. HAV Structure and Genome

**Figure 1:**
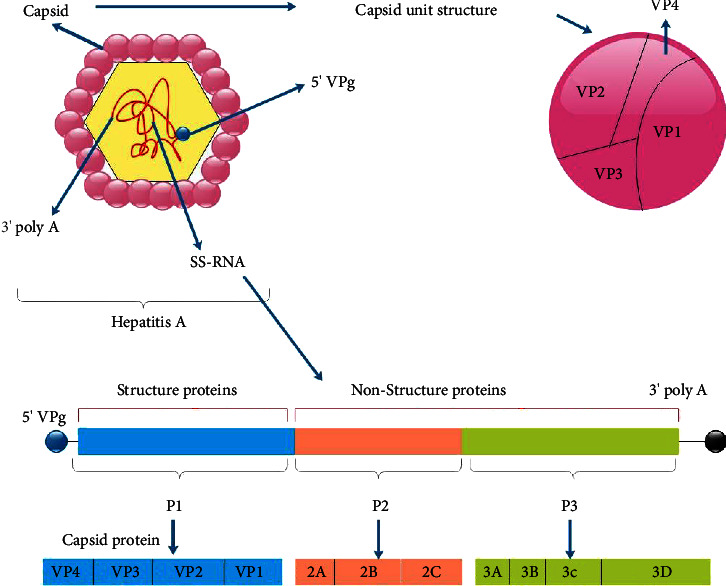
P1 1A-VP4, 1B-VP2, 1C-VP3, and 1D-VP1 are structural protein components making up the capsid polypeptide. P2 comprises of three nonstructural polypeptides: 2A, 2B, and 2C, all required for viral replication. P3 consists of four nonstructural proteins: 3A anchors the replication component to the cell membranes, 3B is a protein that is also called VPg. At the same time, 3C is a cysteine protease that breaks down polypeptides into proteins, and 3D is an RNA polymerase that requires RNA to function.

HAV is a tiny virus with a diameter of 27–32 nm [23]. HAV is a nonenveloped, unusual hepatotropic picornavirus with a 7500 nucleotide positive-strand RNA genome [24–28]. The 5′ ends of the RNA are physically attached to a viral genome-linked protein (VPg) or 3B and also lack a normal cap configuration and a brief untranslated section at the 3′ ends of the genome, which ends in a poly (A) tail. The genome encodes one single large polyprotein, and its translation is regulated by an internal ribosome entrance site (IRES) [3]. There are two noncoding sections at the 5′ and 3′ ends of the HAV genome. The structural proteins are translated from the P1 region, while the nonstructural proteins involved in the reproduction of viruses are encoded in the P2 and P3 regions. The antigenic structure of the capsid is extremely well preserved. As a result, there is only one serotype of human HAV [29]. HAV is made up of four capsid proteins (VP1, VP2, VP3, VP4) and also seven nonstructural polypeptides (2A, 2B, 2C, 3A, 3B, 3C, 3D) [23, 24] (Figure 1).

Infectious viruses come in two types: naked, nonenveloped HAV virions, which are released into the stool, and quasienveloped virions (eHAV), created by seizing control of the host. Endosomal sorting complexes required for transport (ESCRT) by HAV and nonlytic viruses are produced from infected cells, circulating in the bloodstream as a membrane-cloaked complex or in the supernatant of infected cell cultures during acute infection, and then complexes can be produced [30, 31]. The virus present in feces is mostly, if not completely, synthesized in infected liver cells and enters the digestive system after nonlytic release as eHAV over the apical hepatic membrane into the biliary tract. The extreme quantities of bile acids found inside the proximal biliary tract dissolve the membranes surrounding the eHAV capsid, which causes the shedding of bare viruses in the stool. Only a quasienveloped eHAV form is found in the serum or plasma of patients or intentionally infected chimps [31]. Detergent properties of bile acids cause eHAV to lose its membrane in the proximal biliary canaliculi, resulting in nonenveloped virions shed in feces [32]. The capsid is assembled of three major structural proteins: VP0, VP3, and VP1pX [30]. A cellular protease cleaves VP1pX, the largest structural polypeptide, to delete pX from HAV. Previously, pX was misidentified as “2A,” a nonstructural polypeptide found in other picornaviruses that functions as an autocatalytic peptide or viral protease [33]. The 12 amino acids of the N-terminus of pX are essential for effective structural protein synthesis and the formation of the infectious virion. pX appears to be involved in the biogenesis of eHAV virions. For example, the pX polypeptide is only presented in eHAV particles, and the pX component of eHAV particles is observed as an extension of VP1 on the viral capsid's surface. The role of pX in eHAV production, on the other hand, has yet to be fully understood [30]. pX is distinct from any other protein that has been discovered. It is required for assembling the capsid and, most likely, eHAV envelopment, but it is split from the capsid when the membrane is lost [34]. In eHAV virions, the membranes surrounding the capsid lack virally encoded polypeptides, a trait that separates quasienveloped viruses from traditional enveloped virions, and have viral glycoproteins on their capsid [35]. The eHAV membrane shields the capsid from B cell identification and neutralizes antibodies, allowing the virus to propagate more easily within the liver. Naked virions shed in feces are persistent and extremely resistant to drying, enhancing the virus's ability to transmit to naïve hosts through the environment. As a result of this dual lifestyle, HAV has a distinct advantage in terms of survival and transmission within sensitive populations [31]. The tiny viral protein (VP4) is present in the virion, but the unfilled particle only has the uncleaved precursor (VP0) [36]. The smooth particle surface lacks any depressions that could serve as receptor-binding sites. HAV has no pocket factor and can endure extreme levels of heat and pH. Additionally, empty particles are much more resilient than filled ones. The structure-based phylogenetic study classifies HAV between picornaviruses and insect viruses as it takes advantage of the VP2 “domain swap” found in insect picorna-like viruses. HAV's perplexing characteristics may be due to its status as a linkage between “modern” picornaviruses and more “primitive” insect virus ancestors; for example, HAV ability to transcytose from cell to cell [37].

### 2.2. Classification of the HAV

HAV is classified as a member of the *Hepatovirus* genus within the family Picornaviridae, including several other medical and veterinary viruses. Although HAV shares many characteristics with other Picornaviridae family members, its partial nucleotide sequence and numerous unique features set it apart from other picornaviruses, requiring it to be assorted into its genus. HAV strains obtained from nonhuman primates and the ones recently found in bats, hedgehogs, shrews, and rodents appear to share antigenic elements with human HAV and are among the viruses designated as hepatoviruses [25–28]. HAV has one serotype with six genotypes based on examining a 168-nucleotide fragment of the VP1-2A region. Also, genotypes IV–VI cannot infect humans [24, 38]. Genotypes and subtypes tend to show different geographic distributions [39] (Table 2).

### 2.3. HAV Coinfection

Because their entry points are similar, coinfection with HAV and HEV is common. The infection rate of HEV is higher than HAV among acute viral hepatitis cases. In contrast to HEV, HAV exposure is widespread in children. Jaundice, fever, fatigue, and hepatomegaly are common clinical presentations in HAV, HEV, and confection with both viruses in acute viral hepatitis [45]. The double infection can lead to serious complications and increased mortality due to the high risk of acute liver failure in both children and adults. A recent study in India reported that the HEV and HAV coinfection rate is around 6% [46]. Another study conducted in Bangladesh reported that the double infection rate is about 5.3% [47]. The coinfection rate has decreased in recent years, from 2006–2020, and this may be the result of a high rate of HAV vaccination and improved sanitation [45]. Furthermore, Marciano et al. have discovered an unexpectedly high proportion of coinfection of HAV with both HIV and syphilis. HIV-positive individuals had a greater seroprevalence of HAV infection than patients who are HIV-negative. Significant global differences are seen in nations with low and high HAV endemicity. There has been evidence of a high HAV seroprevalence in the HIV community, which has been linked to oral-anal sex, the number of sexual partners, advanced age, and injectable drug use [48].

In addition, a recent syphilis infection was discovered to be related to a hepatitis A infection in a large Taiwanese cohort study conducted during an HAV outbreak in HIV-positive individuals [49]. Likewise, in a study of a recent HAV epidemic in France, 54% of patients had been diagnosed with at least one concurrent sexually transmitted disease (syphilis, chlamydia, and gonococcal infection) [50]. Ghosh et al. [51] discovered dengue fever coinfection in six HAV patients who also had anti-HAV IgM antibodies. Moreover, goal G described the case of a 4-year-old kid who had a partial recovery from an HAV infection but later had dengue fever. Acute fulminant hepatic failure made his situation worse, and he died as a result of a lethal coinfection of dengue and HAV [52]. The concurrent infection of hepatitis A and dengue fever was also discovered in a sixteen-year-old boy, as reported by Bhat et al. from Karnataka, India, with noticeable changes in liver function [53]. Yakoob et al. described a case of dengue fever occurring simultaneously with hepatitis A and E virus infection in Pakistan [54].

### 2.4. HAV Attachment and Entry

TIM1 (T cell immunoglobulin mucin receptor 1), known as HAV cellular receptor 1 (HAVCR1), was once thought to be the HAV receptor [31]. However, a new study denied its essentiality in HAV entry [31]. It has been proposed that gangliosides could be one of the critical molecules promoting HAV entry as they allow the lysosomal escape of HAV particles [55, 56]. Also, the HAV capsid surface is neither as smooth as the foot-and-mouth disease virus capsid nor does it contain the major depressions (canyons) at the bases of the pentamers as seen in enteroviruses like polioviruses that function as receptor-binding sites. In the absence of the canyons, it has been impossible to pinpoint potential receptor-binding sites on the HAV capsid [55, 56]. eHAV lacks embedded glycoproteins as found in traditionally enveloped viruses. Partially due to this anomaly, the mechanistic details of HAV entry within host cells remain unclear. It is thought that the envelope is shed during the interaction of the virus particles with host cells, as the receptor-binding sites are likely a part of the major capsid proteins [56]. The mechanisms of entry into the cell eHAV and naked HAV are different. The entry of eHAV is slow and sensitive to the lysosomal poison chloroquine. In contrast, naked HAV enters rapidly, is resistant to chloroquine, and is not subject to postendocytic neutralization [57, 58].

### 2.5. HAV Replication and Its Inhibitors

**Figure 2:**
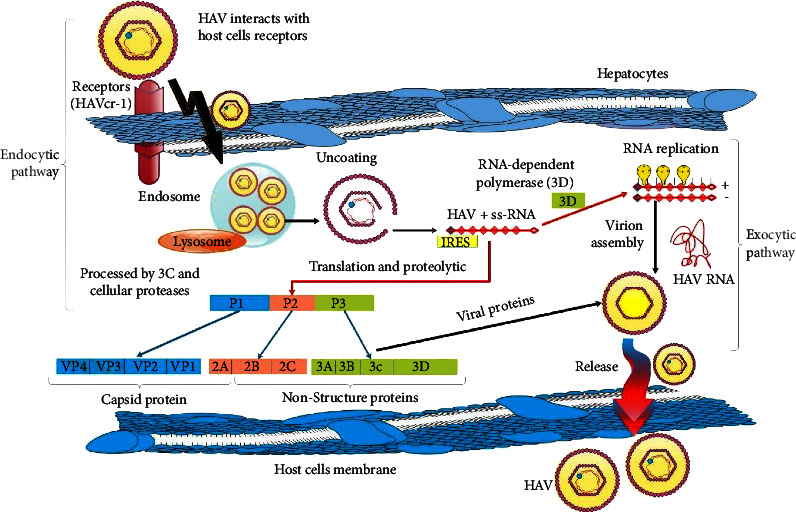
HAV replication cycle. At the hepatocyte's basolateral membrane, the HAV virus interacts with host cell receptors, and its uncoated RNA is released into the cytoplasm. The cap-independent, IRES-driven translation of the positive-strand RNA genome produces polyproteins. Nonstructural proteins involved in genome replication (2B, 2C, 3AB, 3Dpol), the protease (3Cpro), and capsid proteins are produced by proteolytic processing of polyprotein. 2BC causes alterations in intracellular membranes, leading to the formation of a membrane-bound replicase complex that guides the production of a matching minus-strand RNA, which is subsequently employed as a template to make numerous new replicas of the RNA genome. Newly generated positive-strand RNAs are instructed to do more translation or RNA biosynthesis, or they can be packed into capsids to produce intracellular viral offspring. These freshly formed viral components are attracted to multivesicular bodies for eventual egress from infected cells into the biliary canaliculus or hepatic sinusoids via the apical plasma membrane and basolateral plasma membranes, respectively. The RNA genome is exposed after entering the cell, and the host ribosomes attach it to create polysomes. A viral RNA polymerase translation synthesizes viral particles that can be assembled and released into the biliary tree.

HAV replicates in the liver [59] and penetrates host cells by interacting with cell surface molecules, particularly sialic acid and ganglioside, and then uncoating and delivering viral RNA from endosomes to the cytoplasm. The viral polyprotein is synthesized into distinct structural and nonstructural proteins under the supervision of an IRES. HAV then uses cellular membranes to build organelle-like structures in which the viral genome is replicated. When the freshly synthesized genomes are packed, newly synthesized HAV leaves the cells in the quasienveloped virion forms [17, 27] (Figure 2).

HAV is a long-lasting virus with many properties, such as genomic organization and a long replication cycle, distinguishing it from the rest of the mammalian picornaviruses. HAV proteins are produced via cap-independent translation of a singular, long open reading frame under the control of an ineffective upstream IRES. Genome replication is slow and noncytopathic, as in picornaviruses, and transcription is presumably carried out by a uridylated protein primer [60]. Because the IRES nucleotide sequence of HAV is identical among HAV genotypes, it could be a potential antiviral focus. SD1029, AG490, AZD1480, and Janus kinase (JAK) inhibitors decrease host La protein expression, and HAV replication and IRES activity were suppressed. Even though HAV replication seems to be found in the cytoplasm of hepatocytes, human La protein is mainly located in the nucleus and is linked with RNA metabolism, replication, and HAV IRES-dependent translation [61, 62]. Zinc compounds such as zinc oxide, zinc chloride, zinc sulfate, and zinc oxide nanoparticles have anti-HAV features and additive impacts on the anti-HAV properties of interferon [63–65]. Zinc ions are particularly active in toll-like receptor (TLR) signaling pathways. By utilizing a human TLR signaling target RT-PCR array, scientists have investigated its role in human hepatoma cell lines. Zinc chloride also decreases the production of mitogen-activated protein kinase 3 (MAPK3), which might reduce HAV replication in human hepatocytes. Therefore, MAPK3 is an attractive target for therapeutic development since it appears to function as antiviral immunity against HAV infection. Inflammation and endoplasmic reticulum (ER) stress stimulate MAPK3, which phosphorylates and triggers p38 MAPK. Insulin could also activate MAPK3, which is necessary for glucose transporter expression [66]. Zinc sulfate is more likely to boost the anti-HAV action related to interferon-alpha-2a, whereas zinc chloride significantly improves it. Zinc chloride increases mitogen-activated protein kinase 12 (MAPK12) activity while decreasing the expression of six associated genes [63]. Zinc sulfate is an antiviral that slows HAV replication in a dose-dependent manner, reducing HAV replication while increasing the production of GRP78/Bip. GRP78 (glucose-regulated protein 78) is an ER chaperone, participating in unfolded protein response pathways. GRP78 loss increased during HAV replication, resulting in the reduction of ER stress molecules downstream of GRP78. Therefore, GRP78 represents a new hepatocyte-defensive molecule against HAV [67]. Hesperidin and ZnO NPs were tested in vitro against HAV, an example of an RNA virus. As a result, both ZnO NPs and hesperidin have an antiviral effect against HAV [68].

In infected cells with various viruses, including HBV and HCV, the cooxygenase-1 (HO-1) enzyme exhibits antiviral properties [69]. Studies have shown that HO-1 reduces viral replication in HAV-infected cells. As predicted, the HO-1 inducer stimulates HO-1 mRNA and protein expression, reducing RNA viruses and polypeptides in HAV-infected cells in a dose-dependent manner (dose below 50 mM without any cytotoxicity effect). HAV replication was also inhibited by overexpression of the HO-1 protein using a protein expression vector. Although the HO-1 inhibitor, ZnPP-9, was reported to have no impact on HAV replication, it significantly reduced heme-induced antiviral activity in HAV-infected cells. HAV proliferation was also decreased in infected cells by iron (III) chloride (FeCl3), the carbon monoxide-releasing molecule (CORM-3), biliverdin, and HO-1 inducers, as well as andrographolide and cobalt protoporphyrin IX (CoPP), with a dose-dependent effect. These findings suggest that HO-1 inhibits HAV infection in vitro and that its catalytic products have antiviral potential [70].

### 2.6. Morphogenesis and Release

To liberate its membrane-cloaked eHAV form, HAV can seize an apoptosis-linked gene 2-interacting protein X (ALIX)-related exosome-like pathway [30]. HAV replication is tolerated by a wide range of mammalian cell lines (e.g., Huh-7, HepG2, MRC-5, and BSC-1). Although wild-type and low-passage-number types do not cause cytopathology, tiny extracellular vehicles (EVs) that shield the HAV capsid from deactivating antibodies are secreted from such cells without cell lysis [25]. A critical component of the hepatoviral life cycle is the quasienvelopment and extracellular liberation of immature HAV capsids in exosome-like virions. They allow the nonlytic release of the virus and, as a result, noncytopathic replication and latent virus dissemination inside the liver, in addition to protracted infectious virus appearing in stool via the biliary system before the beginning of liver disease [25]. The cellular endosomal sorting complex required for transport (ESCRT) proteins ALIX (ALG-2-interacting protein X) and vacuolar protein sorting 4 homolog B (VPS4B) are involved in the budding of conventionally enveloped viruses, which is required for the biogenesis of quasienveloped eHAV particles. ALIX appears to attach tandem YPX3L “late domains” in VP2, stimulating the budding of assembled capsids into multivesicular bodies (MVBs), leading to the eHAV envelopment and release pathway, which is similar to exosome biogenesis. Because the VP2 late domains are suppressed underneath the naked capsid's surface in the X-ray structure, the capsid may endure considerable conformational alteration with membrane disintegration and pX loss [34].

### 2.7. Clinical Symptoms

HAV infects vulnerable individuals, multiplying in the liver, and the newly reproduced virus is released into the intestines, where it is excreted in feces [25]. Acute hepatitis A has a broad clinical range, from mild instances with no signs to severe cases with acute liver failure and death. However, it does not always lead to chronic hepatitis. The intensity and results of clinical interventions are strongly linked to the age of onset. Nontypical clinical characteristics, such as recurrent hepatitis, prolonged cholestasis, or extrahepatic symptoms, may appear in specific individuals. With supportive treatment, nearly all HAV-infected patients recover on their own. HAV liver damage is assumed to occur after significant damage of infected cells by immune-mediated lysis, which is not directly caused by the virus [71–73]. The incubation period of HAV is typically 14–28 days. Fever, malaise, lack of appetite, diarrhea, nausea, stomach pain, dark-colored urine, and jaundice (yellowing of the skin and whites of the eyes) are all symptoms of hepatitis. Primary HAV infection is usually asymptomatic in children. However, in an adult's body, it can lead to acute hepatitis A (AHA) and severe liver damage. Infected children under the age of six usually show no symptoms of illness (only 10% of them have jaundice). Infection frequently produces more severe symptoms in older children and adults, including jaundice and symptoms like fever and diarrhea (jaundice in more than 70% of the cases). Since HAV infection is generally silent or subclinical, several issues are frequently underestimated [7, 9, 74, 75]. Recurrent infections have been linked to immunological symptoms such as late-onset arthritis, purpura, vasculitis, and myocarditis [76]. Jaundice, anorexia, nausea, vomiting, stomach discomfort, and moderate fever are some of the nonspecific symptoms [43].

HAV can induce severe clinical hepatitis and death in rare cases, but persistent infection or chronic hepatitis is not familiar, even in highly immunocompromised patients [59]. While severe illness (for example, fulminant hepatitis) is hazardous, complications such as nephrotic syndrome, glomerulonephritis, vasculitis, pancreatitis, Guillain–Barré syndrome, thrombocytopenia, or aplastic anemia are uncommon. Still, they can have long-term consequences [26, 77]. Fulminant liver failure, characterized by encephalopathy, jaundice, and an increased international normalized ratio (INR), arises in around 1 percent of HAV infections and is more likely to happen in patients with underlying liver disease or elderlies. In one retrospective study, nearly half of the patients with fulminant liver failure caused by HAV infection needed liver transplantation or died within three weeks after the presentation [78]. Virus-specific antibodies are commonly detected 3–4 weeks after a medically silent disease, which is a sign of both acute liver damage and the illness's remission. Sudden increases in serum alanine transaminase (ALT) activity have been linked to hepatocyte necrosis, apoptosis, and intrahepatic portal inflammatory cell invasions composed of lymphocytes, macrophages, and plasma cells. Although the illness is fatal in a tiny percentage of the infected population, the acute inflammatory phase of the disease is usually brief and self-limiting. After the emergence of antibodies, fecal release and HAV replication in the liver decrease quickly, and the ALT level reaches its baseline in several weeks [79]. Chronic infections are uncommon, and neutralizing anti-HAV antibodies gives long-term immunity from illness recurrence [25]. Infected individuals gain immunity after an acute hepatitis A infection. A tiny population of cases (about 10%) will experience relapse. HAV relapse is most likely between 1 and 3 months following the first infection, but it can occur up to 1 year later. Relapses are infections that are resolved with clinical and laboratory discoveries and recurring after weeks or months. Serum IgM antibodies are frequently positive during a relapse. They are usually less severe than during acute infection, with aminotransferases occasionally exceeding 1,000 IU/dL and liver enzymes exhibiting a cholestatic pattern. HAV extrahepatic symptoms such as a pruritic rash and arthralgia are more likely to appear during relapses. Any patient with increased liver enzymes ought to be suspected, which may be verified by hepatitis A antibody tests [76, 80].

### 2.8. HAV Diagnosis

**Figure 3:**
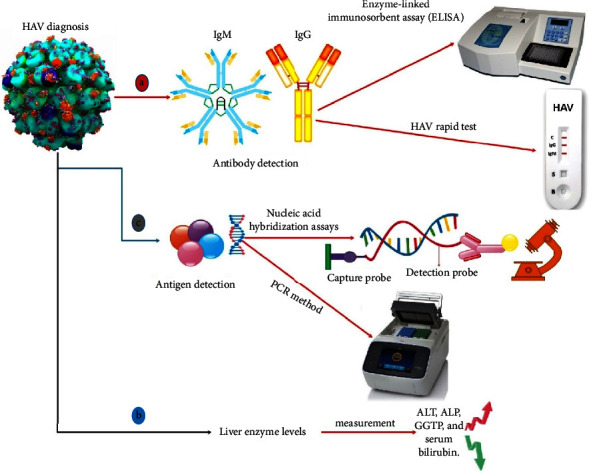
Different methods can be used to diagnose HAV infection, such as (a) specific diagnosis based on antibody detection: IgM antibodies are presented in the early stages, whereas IgG antibodies can persist for up to 6 months after infection. ELISA and immunochromatographic-based assays are used to differentiate between antibodies. Additional tests include (b) checking the level of liver enzymes and serum bilirubin and (c) molecular methods such as PCR to detect the virus genome.

In the laboratory, clinical specimens such as blood, feces, bile, liver biopsy, and serum are utilized to detect HAV. The following items are some of the most common procedures for hepatitis A laboratory diagnosis:

(1) Antibody detection: HAV-specific IgM antibodies are typically found in the earliest stage of disease and can last for six months after infection. IgG antibodies can last for many years. Enzyme-linked immunosorbent assay (ELISA) is used to distinguish antibodies. Rapid tests (based on immunochromatographic technologies) can also be used to detect antibodies. (2) Liver enzyme levels: measurement of liver enzyme levels such as ALT, alkaline phosphatase (ALP), gamma-glutamyl transpeptidase (GGTP), and serum bilirubin. (3) Antigen detection: PCR and nucleic acid hybridization assays are used to identify antigens. Also, conventional testing for HAV relies on anti-HAV IgM seropositivity. However, studies estimate that 10–30% of patients may not be diagnosed by serology. Molecular assays that can directly detect viral nucleic acids have the potential to improve diagnosis (such as real-time PCR), which is key to preventing the spread of infections [81–87] (Figure 3). Antibodies are the body's natural protection against infection, and their level of HAV in the blood is used to detect whether a patient has an ongoing or prior infection. In 2–3 weeks following the first infection, patients produce IgM antibodies against HAV. Circulating IgM antibodies signal an active infection lasting up to six months. 1–2 weeks after IgM antibodies secretion, IgG antibodies emerge and approve lifelong immunization against HAV. A high level of IgG antibodies refers to a previous illness or vaccination. HAV IgM, HAV genetic diversity, and genotypes can be detected in serum using ELISA test, PCR sequencing, and phylogenetic analysis, respectively [84].

HAV identification in food has been accomplished by utilizing molecular tests with fit-for-purpose primers to provide 100% specificity. Although conventional PCR is still valid since more oversized PCR products are generated to validate the target's specificity, quantitative RT-PCR (RT-qPCR) technologies have positively altered viral identification in medical and environmental samples. The high sensitivity of RT-qPCR, its low cost, and the increased specificity achieved by using fluorescent probes have made it the gold standard for HAV detection in food. RT-qPCR, in particular, allows for quantitative viral detection for threat risk assessment analysis and is applied to enhance preventative measures and food-related laws [86, 88]. While developing a robust cell culture method for assessing HAV infectivity in food might be a huge step, RT-qPCR is a quick and low-cost technique that offers a more precise indication of the risk related to infected food and water [88]. Various approaches, such as cell culture, RT-PCR, biosensors, and other tests, are utilized to determine viruses. However, researchers use molecular fluorescence sensors to determine macromolecules by altering the color of a solution under UV light at 365 nm with the naked eye. This diagnostic technique is carried out to identify HAV and HBV simultaneously [89].

### 2.9. Diagnose Errors

When people are infected with HAV, it is medically impossible to distinguish the infection from other types of acute viral hepatitis, and the disease is generally mild and self-limiting. Elderly or immunocompromised people suffering from chronic liver disease or other underlying health problems are in danger of developing symptoms [8]. Environmental factors, such as viral infections, particularly HAV, are thought to be a possible cause of autoimmune hepatitis (AIH) [90]. AIH is triggered by the immune system and affects people of all ages. For years, the association between HAV and AIH was misunderstood because the cross-reactivity of antibodies might result in a false-positive anti-HAV IgM outcome in autoimmune disorders and chronic and acute infections. As a result, IgM testing should not be prescribed as the only way of identifying acute HAV infection. To validate the diagnosis, HAV nucleic acid amplification assays might be helpful in the diagnostic process to confirm acute hepatitis A, especially in individuals who test positive for IgM anti-HAV but have an average or low signal-to-cutoff (S/CO) ratio. False anti-HAV IgM serological findings might cause misdiagnosis or early diagnosis cancellation. Therefore, detecting acute HAV infection exclusively by anti-HAV IgM is insufficient. HAV nucleic acid studies could be conducted more widely, especially in individuals with lower cutoff values for anti-HAV IgM [91].

Guillain–Barré syndrome (GBS) is a peripheral demyelinating illness frequently followed by a gastrointestinal infection. The infectious cause is mainly bacterial or viral, with *Campylobacter*, influenza virus, Ebstein–Barr virus, and cytomegalovirus (CMV) infections being the most prevalent [92]. Even though HAV is not traditionally linked to GBS, researchers have discovered that GBS is caused by HAV infrequently. In a case report study, IgM and IgG antibody levels seem to be a sign of relapsing hepatitis A. However, later on, the patient was diagnosed with GBS and was treated with intravenous immunoglobulin [92, 93]. Although IgM anti-HAV persists after HAV infection resolution, other circumstances can lead to IgM anti-HAV false-positive findings in routine tests. IgM lasts up to 6 months on average, but it can last for a year after the infection has resolved, possibly causing an inaccurate identification of acute hepatitis A [94]. False-positive findings were also found, particularly in individuals whose symptoms did not match the clinical criteria for acute hepatitis A. In situations of polyclonal activation of B lymphocytes, the production of IgM antibodies is promoted in HAV seropositive people. Cross-reacting antibodies have also been documented as a source of false-positive anti-HAV findings in patients enduring acute or chronic diseases and autoimmune disorders [95]. Also, current HAV exposure diagnostic tests, such as the Abbott HAV antibody test, identify antibodies primarily to structural polypeptides and hence cannot discriminate between natural infection and vaccination [96]. As a result, the presence of anti-HAV IgG and the absence of anti-HAV IgM indicate immunity via previous infection or vaccination [78].

### 2.10. Screening Errors

During the last few decades, blood safety has grown in importance as a means of preventing diseases associated with transfusions [97]. Transfusion-associated hepatitis A (TAHA) may be underrecognized. Although TAHA rarely results in severe infection, the risk it creates of secondary transmission, especially within the hospital setting, is not inconsequential [98]. Hughes et al. reported TAHA in 2014. A donor developed symptoms of hepatitis 20 days after donation. The recipient of the plasma, a 15-month-old female, tested positive for immunoglobulin M antibody to hepatitis A virus 43 days after the transfusion. The recipient displayed mild, nonspecific symptoms approximately two weeks after the transfusion [98]. For the first time, Hettmann et al. reported the TAHA outbreak in Hungary in 2014, involving 5 cases. A 41-year-old man, who was a regular apheresis donor, informed the Hungarian National Blood Transfusion Service (HNBTS) of his HAV infection 18 days after his last donation. A 6-year-old boy recipient and his grandmother, the other 65-year-old woman recipient, and one of the nurses in the hospital tested positive for HAV infection [99]. Nevertheless, in 2018, Lefeuvre et al. reported a TAHA infection in an immunocompromised patient in France [100]. Parenteral hepatitis A transmission is uncommon, but it can happen during viremia [101]. However, because of the sporadic nature of acute HAV infections in donors, the short viremia phase, and the absence of chronic carriers, HAV RNA screening is rarely reported [100]. Therefore, increased vaccination rates and the immunization of frequent blood donors may lower the risk of transfusion-transmitted hepatitis A infections [99].

### 2.11. Vaccination

Infection with HAV can cause the development of antibodies against the virus's structural and nonstructural polypeptides. In contrast, an inactivated or attenuated HAV vaccine produces antibodies primarily against structural proteins, with little or no antibody production against nonstructural proteins [96]. Hepatitis A vaccination comes in two varieties: one type of vaccination is administered in two doses, six months apart, and both injections are required for long-term hepatitis A protection. The other type is a dual-protection vaccination, which protects against HAV and HBV. The single-antigen vaccinations are Havrix and Vaqta, as well as the combination vaccine Twinrix (containing both HAV and HBV antigens). Anyone above the age of 18 can receive the combination vaccination, which is administered in three doses over six months. HAV and HBV require all three doses to provide long-term protection obtained after receiving the whole vaccination series [44]. Another vaccination used in China is the live attenuated HAV vaccine (HA-L), which is routinely used in the Chinese National Immunization Program (NIP). Mutation shifts and subsequent infections of the live vaccination virus strain are one of the major downsides of HA-L [24].

**Figure 4:**
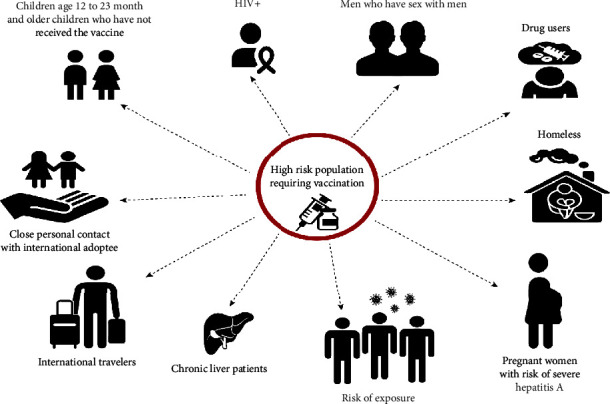
The Centers for Disease Control and Prevention (CDC) recommend hepatitis A vaccination for different groups of populations, including one-year-old children, all children and teens up to age 18 who have not previously been vaccinated, certain children aged 6–11 months who are traveling outside of their countries, all adults who are at risk, or adults who do not have a risk factor but want to avoid hepatitis A infection.

People at risk of contracting the virus should be vaccinated. People with homosexual tendencies or travelers to countries with high or moderate HAV rates and those consuming illegal substances or suffering from chronic liver disease, as well as those in jail, emergency rooms, syringe exchange programs, and drug treatment facilities, are among the most vulnerable [75, 102, 103]. For people with blood clotting factor abnormalities and hemophiliac patients getting plasma-derived therapy, serology control during follow-up should be offered, potentially within two years, because HAV titers may rapidly fall. Anyone who deals with HAV-infected primates or HAV in a research lab environment is also in danger [104–106]. All human immunodeficiency virus (HIV)-infected patients with no anti-HAV antibodies are at risk of HAV infection or severe disease and should get vaccinated [106]. The hepatitis A vaccination is suggested for all children aged one or above. However, the vaccine's lifetime preservation is uncertain, and protection throughout maturity is critical to avoid hepatitis behindhand [107]. Even in industrialized nations, HAV is still one of the important causes of death (Figure 4).

The existing HAV vaccines depend on generating wild-type or attenuated virus in cell culture, which increases production costs [108]. The creation of viral structural proteins in recombinant form in commonly accessible expression systems is a goal for the development of cheaper subunit vaccines or techniques for antibody-based diagnostics. Nain et al. examined many ways in the *Escherichia coli* expression system for recombinant synthesis of one of the vital capsid proteins, VP1, from HAV. VP1 oligomers produced in a bacterial expression system can be used to study the molecular mechanism of HAV capsid formation and may have biological applications in preventing HAV infections [56]. Nanoparticles have been clinically approved as vaccines for infectious diseases such as organic nanoparticles. In the case of HAV, the first licensed liposomal vaccination product for treating HAV was the Epaxal vaccine. Crucell Berna Biotech designed this vaccine, which contains a formalin-deactivated hepatitis A (RG-SB strain) antigen and viral envelope glycoprotein inside its phospholipid bilayer. The lipid components of the Epaxal vaccine, dioleoyl phosphatidylethanolamine (DOPE) and 1,2-dioleoyl-sn-glycero-3-phosphocholine (DOPC), enhanced the absorption of HAV antigen to immunocompetent cells and provided immunogenicity from HAV injection for more than 20 years following two booster doses [109]. Numerous parameters, such as CD4 cell count, CD4/CD8 ratio, viral load, concurrent hepatitis C infection, and gender differences, have been proposed as potential factors in the serum conversion rate following HAV immunization [106]. In recent studies, the immune response to the HAV vaccine in youngsters with periodic fever, aphthous stomatitis, pharyngitis, and adenitis (PFAPA) has been proven to be highly immunogenic and tolerated [110]. Immune globulin is an antibody-containing material derived from human blood plasma. Immune globulin, unlike the HAV vaccination, does not give long-term protection against the infection [11]. Long-term immunity is obtained by vaccination or past exposure to hepatitis A. When the exposure occurred fewer than 14 days ago, immunoglobulin may be used as a postexposure prophylactic [43]. Anti-HAV IgG generated in response to HAV infection lasts a lifetime and avoids reinfection. Moreover, long-term immunity is conferred by IgG anti-HAV generated after vaccination [8]. Serology should be recommended before immunization in places with intermediate/high HAV seroprevalence. In populations receiving a transplant, HAV vaccination is the cause of antibody interference. Alternative HAV immunization regimens should be tested in a prospective experiment [111].

### 2.12. Prevention

Although common sexually transmitted disease (STD) prevention techniques (e.g., condom usage or personal hygiene guidelines) are insufficient to avoid HAV transmission, they are a vital aspect of education for the MSM and the population who are engaged with high-risk sexual behaviors. Functional vaccination is available and safe, so its usage is advised to avoid completely transmitted infections and outbreaks [7]. Additionally, the HAV can be eliminated by heating foods to 185 Fahrenheit for at least 1 minute, chlorinating contaminated water, or washing contaminated surfaces with a bleach-water solution [78].

One approach to avoiding HAV infection is nutrition, which can inhibit HAV growth and replication. As previously stated, higher GRP78 expression inhibits HAV replication [112]. Japanese miso extracts obtained from koji rice boost GRP78 expression. Human hepatoma Huh-7 cells and human hepatocyte PBX cells were used to test the efficacy of Japanese miso extracts as antiviral agents against HAV. Japanese miso extracts boosted GRP78 expression and inhibited HAV replication in human hepatocytes. These findings reveal that Japanese miso extracts can partially modulate GRP78 expression and function as antivirals against HAV infection in either an additive or synergistic manner. As a result, miso extracts can be used as a dietary supplement to treat severe hepatitis A [113].

### 2.13. Treatment Approaches

Despite the accessibility and effectiveness of hepatitis A vaccinations, hepatitis A outbreaks continue to be reported globally. Creating antiviral medication is significantly challenging. Antiviral-based medication may be helpful in preventing and treating fulminant hepatitis, shortening the infective phase, and decreasing the severity of symptoms, hence lowering the likelihood of outbreaks and the transmission of vaccine-escape mutations [114, 115]. Direct-acting antivirals (DAAs) and host-targeting agents (HTAs) are the two types of antiviral medications examined to treat HAV. Protease inhibitors, a polymerase inhibitor, and IRES inhibitors are all components of DAAs that particularly target HAV. DAAs do not have any of the side effects of interferon such as depression, hematologic side effects, or flu-like syndrome. HTAs have pan-genotypic antiviral activity and substantial genetic barriers to resistance. HTAs often work in concert with DAAs since their mechanisms of action are complimentary to those of DAAs [116] (Table 3). Numerous antiviral medications have been tested in vitro and in vivo, however, there are currently no known cures for hepatitis A. Further investigation is needed to ensure these agents' safety and efficiency [114]. Fortunately, HAV infections are typically self-limited, and supportive care is a part of treatment process. It is also advised to avoid unnecessary medication [16].

## 3. Conclusion

As addressed in this article, HAV is still a serious concern in developing countries. The epidemiology of HAV infection should be monitored constantly to reduce the risks of any outbreaks, which could be achieved by undertaking cost-effectiveness analyses of HAV immunization techniques. Also, immunization has to be considered an important step, and this system will enable the early detection of epidemiologic transitions and the implementation of preventative efforts before HAV infection becomes a public health issue. Owing to the importance of this virus, further relevant studies are crucial to revealing unknown aspects of the HAV entrance mechanism to amend the lack of appropriate therapeutic medication.

## Figures and Tables

**Table 1:** HAV distribution rates per 100,000 population by age groups in the recent years based on CDC reports.

Age	Years				
	2016	2017	2018	2019	2020
0–19	0.4	0.3	0.7	0.9	0.3
20–39	1.8	3.5	15.9	22.4	10.9
40–59	1.5	2.7	10.1	16.6	9.5
≥60	0.6	0.7	1.4	2.3	1.4

**Table 2:** HAV genotypes and subtypes and their geographical distributions.

Hepatitis A virus genotypes	Hepatitis A virus subtypes	Geographical distribution	Comments	References
Genotype I	A, B, C	Subgenotype IA was reported in China, Japan, Turkey, Russia, Hungary, the United States of America (US), and Germany	(i) Infecting humans, the most frequent genotype is the I genotype. The IA subgenotype is more frequent than IB	[22, 24, 38, 40–42]
			(ii) Genotype IA was most common among strains tested in US outbreak investigations and surveillance during 1996–2015	
		Subgenotype IB has been reported in Turkey, the Netherlands, Hungary, France, Italy, Bulgaria, and Egypt	(iii) Genotype IB gained prominence during 2016–2019 person-to-person multistate outbreaks	
			(iv) Subgenotype IB is the most prevalent genotype in Turkey	
			(v) The cell culture-adapted viruses most commonly used are variants of the Australian strain HM175 (genotype IB) and the German strain GBM (genotype IA)	

Genotype II	A, B	Until today, only a few cases of genotype II have been reported	Infect humans	[22, 24, 29, 38, 40, 43, 44]
		With potential African origin of IIA strains and occasionally found in France		

Genotype III	A, B	Genotype III has a worldwide distribution pattern. Subgenotype IIIB is mainly seen in Japan	Infect humans	[22, 24, 29, 38, 40, 41, 43, 44]
		Subgenotype IIIA has been reported in Iran, The Netherlands, South Korea, Japan, Afghanistan, Turkey, and India		

**Table 3:** Different types of antivirals previously tested against hepatitis A.

Antiviral agents against HAV	Types	Description	Ref.
*HATs*			
Interferons	(i) Interferon-alpha (IFN-*α*)	(i) IFN-*α* has antiviral activity against HAV replication	[117–121]
	(ii) Interferon-gamma (IFN-*γ*)	(ii) Its use is unsafe for severe HAV infections, including fulminant hepatitis	
	(iii) Interferon-lambda (IL-29, IL-28A and B)	(iii) Recombinant IFN-*γ* displays antiviral activity against chronic HAV infection	
		(iv) IL-29 and IL-28A inhibit HAV IRES-mediated translation	
		(v) Compared to IFN-*α*, it had fewer side effects, such as hematological cytotoxicities or depression	
Ribavirin		(i) Acts against RNA and DNA viruses	[122, 123]
		(ii) Moderately affects HAV replication in cell culture	
Amantadine		(i) Inhibits viral antigen synthesis, HAV IRES-mediated translation, and HAV replication	[117, 124]
		(ii) Its effects may be strain-dependent	
		(iii) Stronger inhibitory effects on HAV replication were seen when amantadine was combined with IFN-*α* or IL-29	
Agents against host enzymes and cellular factors	(i) Autoantigen la	(i) These proteins may interact with HAV IRES RNA and might be associated with HAV replication and IRES-mediated translation	[62, 125–128]
	(ii) GAPDH		
	(iii) Polypyrimidine tract-binding protein		
	(iv) Poly(C) binding protein 2		
	(v) Polyadenylate-bindingprotein-1 (PABP)		
	(vi) Eukaryotic translation initiation factor 4E (eIF4E) an4G (eIF4G)		
	(vii) Janus kinase (JAK) inhibitor		

*DAAs*			
Cysteine protease inhibitors	(i) Peptide aldehyde	(i) Play a crucial part in the HAV polyprotein's processing, thus affecting HAV replication	[129–132]
	(ii) A peptidyl monofluoromethyl ketone (peptidyl-FMK)		
	(iii) Beta-lactones		
	(iv) Hexanucleotide (G(5)T)		
siRNAs	(i) siRNAs against the HAV 2C- and 3D-coding regions	(i) siRNAs generally knock down target genes and prevent them from producing a functional protein	[133–136]
		(ii) This group of siRNAs acts against HAV nonstructural protein-coding regions related to HAV replicon replication	
	(ii) RNase III endoribonuclease-prepared siRNAs (esiRNAs) and some short hairpin RNAs (shRNAs)	(iii) They target HAV IRES and suppress HAV IRES-mediated translation	
		(iv) Compared to a single transfection, subsequent siRNA transfections targeting different HAV genome sequences may have a more effective and long-lasting silencing effect	

## Data Availability

The data supporting the findings of this study are available from the corresponding author upon request.

## Abbreviation

HAV: Hepatitis A virus
HBV: Hepatitis B virus
HCV: Hepatitis C virus
HDV: Hepatitis D virus
HEV: Hepatitis E virus
WHO: World Health Organization
CDC: Centers for Disease Control and Prevention
FRHK4: Fetal rhesus monkey kidney cell line
VPg: Viral genome-linked protein
IRES: Internal ribosome entrance site
eHAV: Quasienveloped virions
FeCl_3_: Iron (III) chloride
CORM-3: Carbon monoxide-releasing molecule
CoPP: Cobalt protoporphyrin IX
ALIX: Apoptosis-linked gene 2-interacting protein X
ESCRT: Endosomal sorting complexes required for transport
JAK: Janus kinase
TLR: Toll-like receptor
GRP78: Glucose-regulated protein 78
MAPK3: Mitogen-activated protein kinase 3
ER: Endoplasmic reticulum
EVs: Extracellular vehicles
MAPK12: Mitogen-activated protein kinase 12
VPS4B: Vacuolar protein sorting 4 homolog B
MVBs: Multivesicular bodies
ALT: Alanine aminotransferase
HAI: Inactivated HA vaccine
HA-L: Live attenuated HAV vaccine
AHA: Acute hepatitis A
INR: Increased international normalized ratio
ELISA: Enzyme-linked immunosorbent assay
ALIX: ALG-2-interacting protein X
RT-qPCR: Quantitative RT-PCR
ALT: Alanine transaminase
ALP: Alkaline phosphatase
GGTP: Gamma-glutamyl transpeptidase
AIH0: Autoimmune hepatitis
NIP: National Immunization Program
HIV: Human immunodeficiency virus
STD: Sexually transmitted disease
GBS: Guillain–Barré syndrome
CMV: Cytomegalovirus
MSM: Men who have sex with men
S/CO: Signal-to-cutoff
DAAs: Direct-acting antivirals
HTAs: Host-targeting agents.
